# One‐minute heart rate variability – an adjunct for airway obstruction identification

**DOI:** 10.14814/phy2.13948

**Published:** 2019-01-10

**Authors:** Amit Lehavi, Neta Golomb, Ronit Leiba, Yeshayahu (Shai) Katz, Aeyal Raz

**Affiliations:** ^1^ Department of Anesthesiology Rambam Health Care Campus the Ruth and Bruce Rappaport Faculty of Medicine Technion – Israel Institute of Technology Haifa Israel; ^2^ Department of Epidemiology Rambam Health Care Campus the Ruth and Bruce Rappaport Faculty of Medicine Technion – Israel Institute of Technology Haifa Israel; ^3^ Department of Anesthesiology University of Wisconsin Madison Wisconsin

**Keywords:** Airway obstruction, cardio‐respiratory interaction, electrocardiography, heart rate variability

## Abstract

Heart rate variability (HRV) reflects cardiac and autonomic nervous system activity. It is usually measured over a relatively prolonged period and presented using multiple parameters. Here, we studied rapid HRV changes during airway obstruction using a short (1 min) sampling window. Forty healthy volunteers underwent a trial of obstructed breathing. Heart rate was recorded during three consecutive sets comprised of 1‐min control followed by 1 min of obstructed breathing, with 1 min of rest between sets. Time and frequency domain analysis were used to compare HRV during control versus obstructed breathing. **C**ompared with control, HRV intensely increased during obstructed breathing: R‐R intervals (time between consecutive *R* waves) standard deviation increased from 65 to 108 msec (*P* < 0.0001), root mean square of successive R‐R interval from 61 to 82 msec (*P* = 0.001), number of pairs of successive R‐R intervals that differ by more than 50 msec (NN50) from 16.5 to 25.3 events (*P* < 0.0001), and proportion of NN50 divided by total number of R‐R intervals from 26.6 to 35.1% (*P* = 0.001). Low frequency power increased by more than fourfold (*P* < 0.0001), allowing 90% sensitivity and 75% specificity for identifying airway obstruction (ROC area 0.88, *P* < 0.0001). We observed a rapid intense increase in HRV during obstructed breathing, significant enough to detect during a short 1‐min sampling window. These findings suggest that HRV may be useful for rapid detection of airway obstruction, especially in situations where end‐tidal CO_2_ monitoring is not optimal, such as during partial airway obstruction.

## Introduction

Airway obstruction, complete or partial, is a critical event during sedation and anesthesia. It requires rapid identification and intervention to avoid serious morbidity and mortality. The current gold standard monitoring airway patency is end‐tidal CO_2_. This is useful, effective, and reliable, and considered a standard of care during sedation and anesthesia by many anesthesia societies (Whitaker and Benson 2016). However, end‐tidal CO_2_ which is accurate and reliable for identifying complete airway obstruction and apnea, does not identify partial airway obstruction, and may fail to identify respiratory adverse events (Pekdemir et al. 2013; Ebert et al. 2017). This could happen during sedation with spontaneous breathing, as patients may switch between nasal and mouth breathing, precluding reliable end‐tidal CO_2_ monitoring or causing multiple false alarms that interfere with effective monitoring. Moreover, this monitor is insensitive to partial airway obstruction. Therefore, so far identifying partial airway obstruction remains a clinical diagnosis. Airway obstruction during anesthesia may gradually develop, and become apparent only when it is severe, often causing a medical emergency that can rapidly deteriorate to a catastrophe. Any delay in its detection by current clinical anesthesia monitors may result in a severe outcome. Thus, adjuvant monitors that will identify airway obstruction early on, and will not be affected by the same pitfalls as end‐tidal CO_2_ may greatly improve the patient's safety, and provide a warning sign for the clinician allowing early intervention and prevention of such an event.

HRV is a mathematical measure of physiological activity variation derived from the time interval between heartbeats which results from a delicate interplay of the sympathetic and parasympathetic nervous systems. HRV was shown to be affected by emotional states, cardiac conditions, and other factors (Rajendra Acharya et al. 2006).

Heart rate variability (HRV) is modulated by the respiratory system (Elghozi et al. 1991; Vidigal et al. 2016), as the cardiac preload is modified via intrathoracic pressure which is respiratory effort dependent. Pulmonary disorders, such as asthma (Garrard et al. 1992; Gupta et al. 2012) and obstructive sleep apnea (OSA) (Khoo et al. 1999; Gula et al. 2003; Kufoy et al. 2012; Brzecka et al. 2015) were shown to modulate HRV. Airway obstruction leads to significant modulation of the intrathoracic pressures in spontaneously breathing patients. It had been previously shown that HRV was effected by airway obstruction under anesthesia (Arai et al. 2007). Previous guidelines recommended 5 min as a minimal electrocardiogram (ECG) recording time for short‐term HRV measurements (Task Force of the European Society of Cardiology and the North American Society of Pacing and Electrophysiology, 1996), a delay too long for timely detection of airway obstruction.

In this work, we studied heart rate variability (HRV) as an adjunct to end‐tidal CO_2_ monitoring. This cheap, simple, and noninvasive technique can be easily added to a standard monitoring to help identifying critical airway events. We systematically evaluated the effect of simulated severe airway obstruction on HRV parameters calculated over 1‐min interval. This was aimed to set basis for rapid analysis and detection of airway obstruction.

## Materials and Methods

Following approval (0031‐15‐RMB; National Institutes of Health trial number NCT03733704) by the Institutional Review Board of Rambam Health Care Campus, Haifa, Israel (Chairperson Prof N. Krivoy) on 29 March 2015, the study was performed in a prospective cross‐over, longitudinal cohort manner. Healthy volunteers aged between 20 and 40 years were included in this study. Each participant signed an informed consent. Exclusion criteria were respiratory or cardiac pathology, diabetes, a body mass index lower than 18 or higher than 26 kg m^−2^, chronic medical conditions with significant involvement of the sympathetic or parasympathetic systems (e.g., diabetes), obstructive sleep apnea, use of medications with either cardiovascular or respiratory effects, and ECG abnormalities including heart rate other than sinus rhythm, atrioventricular conduction blocks, intrafascicular conduction delays, or prolonged QT interval (the time between consecutive *Q* and *T* waves on the electrocardiogram).

Initially, each volunteer underwent an ultrasound evaluation (SonoSite™ M‐Turbo™ ultrasound machine, SonoSite, Bothell, WA) of the lungs using a high‐frequency linear probe at mid‐clavicular lines to exclude pneumothorax, an apical view of the heart using a curve‐linear probe to exclude pericardial effusion. A 12 lead ECG was obtained and analyzed to exclude rate or conduction abnormalities. The ECG and ultrasound evaluation were performed and interpreted by an anesthesiologist from the study team (either A.L or N.G.). Noninvasive blood pressure and oxygen saturation were recorded in all subjects throughout the study to identify and prevent any complication.

A three lead ECG and spirometry were obtained in supine position with the upper body raised by 30 degrees. Data were collected using a Datex AS/3 monitor (Datex Ohmeda Medical Equipment, GE Healthcare). The data were recorded using a digital to analog acquisition card (NI‐6008, National Instruments™, Austin, TX) and a Biosignal Logger of National Instruments™ Biomedical Workbench ™ at a sampling rate of 500 Hertz (Hz). All experiments were performed at the same time of day (early afternoon), and under the same conditions (same place and experimental setup). Volunteers were instructed to refrain from smoking for 4 hours prior to participating in the study.

**Figure 1:**
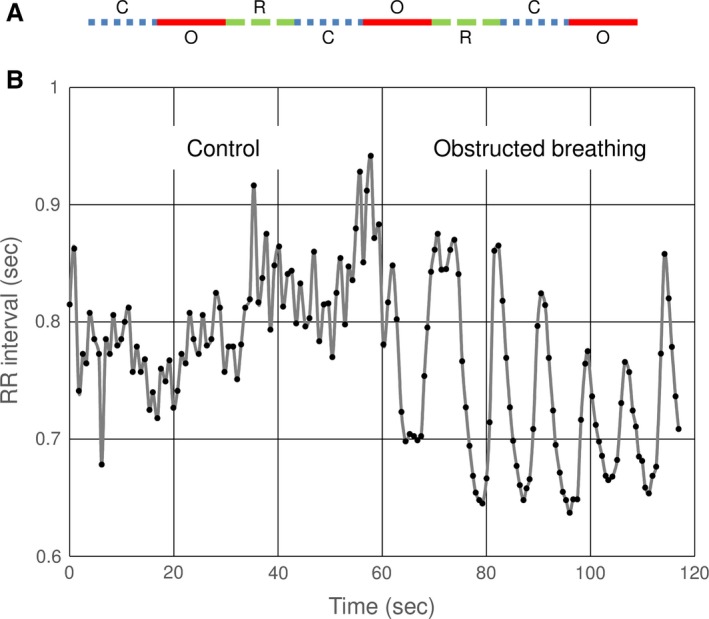
(A) Experimental paradigm. Each volunteer performed three sets of 1 min of control (normal, unobstructed breathing) marked by a blue dashed line, immediately followed by `1 min of obstructed breathing, marked by a solid red line. Following each set, the volunteers were allowed at least 1 min of rest, marked by a widely dashed green line, to recover and return to their baseline breathing before the next set. (B) An example of the change in R‐R intervals during 1 min of control (normal, unobstructed) breathing immediately followed by 1 min of obstructed breathing. It can be seen that during the obstructed breathing the heart rate variability increases, and that the heart rate oscillates in synchrony with the respiratory effort (i.e., with the intrathoracic pressure). C , control; O, obstructed breathing; R, rest; R‐R interval, time between two consecutive *R* waves on the electrocardiogram.

The airway obstruction was simulated by an 18 cm long, 4 mm internal diameter endotracheal tube, connected to a spirometry adaptor and an antimicrobial filter. During the obstructed breathing phase, the volunteers were directed to seal their lips tightly around the filter to prevent air leak and encouraged to reach a peak expiratory pressure of 30–40 cm H_2_O, using the instantaneous display on the spirometry monitor. Three sets were recorded for each volunteer; each set was comprised of 1 min of normal unobstructed breathing that served as control, immediately followed by 1 min of obstructed breathing. Following each set, the volunteers were allowed at least 1 min of rest period to recover and return to their baseline breathing before the next set (Fig 1A).

### HRV analysis

A detailed description of the means to measure and evaluate the significance of HRV can be found in the European Society of Cardiology and the North American Society of Pacing Electrophysiology Task Force on heart rate variability, standards of measurement and physiological interpretation and clinical use guidelines (Task Force of the European Society of Cardiology and the North American Society of Pacing and Electrophysiology, 1996). Briefly, the raw ECG signal was preprocessed (including high pass filtering to remove Baseline wandering and ECG feature identification) and the R‐R intervals were extracted from the raw ECG signal using ECG Features Extractor of National Instruments™ Biomedical Workbench™ with threshold adjust factor of 0.1, a rough highest heart rate of 60 beats min^−1^, *R* waves frequency of 10–25 Hz and middle *R* waves onset and offset.

We evaluated HRV using time and frequency domain methods. Time domain measures are based on the statistical analysis of the time interval between two adjacent *R* waves on the electrocardiogram complexes, referred to as R‐R intervals (time between two consecutive *R* waves on the electrocardiogram). R‐R intervals standard deviation (SD), root mean square of successive differences (RMSSD) between adjacent R‐R intervals, number of pairs of successive R‐R intervals that differ by more than 50 msec (NN50) and proportion of NN50 divided by total number of R‐R intervals (pNN50) are routinely used to quantify HRV. Frequency domain employ mathematical manipulation to the signal, such as the fast Fourier transform (FFT), which converts the time function into a sum of sine waves of different frequencies. These are used to calculate the power spectral density in very low (VLF), low (LF) and high frequency (HF) ranges and provide a quantification of the physiological HRV‐related effects. In this report, we did not include the VLF, as this value cannot be reliably measured with a brief measurement window of 1 min. We normalized the HF power and LF power to the total power that is, LFnorm = (LF*100/LF + HF) and HFnorm = (HF*100/LF + HF). The normalized values are referred to as HFnorm and LFnorm.

HRV parameters were calculated over 1‐min sampling window using the Heart Rate Variability Analyzer of National Instruments™ Biomedical Workbench™. Fast Fourier analysis was employed using a Hanning Window of 1024 samples with a 50% overlap, and with a 2 Hz interpolation rate and 1024 frequency bins. As suggested in the literature for the power spectral density calculation, HF was defined as 0.15–0.4 Hz, LF as 0.04–0.15 Hz, and VLF as below 0.04 Hz (Task Force of the European Society of Cardiology and the North American Society of Pacing and Electrophysiology, 1996).

### Respiratory rate calculation

To evaluate the changes in respiration during the obstructed breathing, we calculated the respiratory rate during the control and obstructed breathing periods. We used the raw ECG traces to calculate the respiratory rate (Sinnecker et al. 2014). This was done primarily based on the *R* wave amplitude and calculating the number of local peaks in the sampling window. To obtain meaningful results, we chose the control and the obstructed breathing segments with the most obvious changes in the *R* wave amplitude. Using this methodology, we could reliably evaluate the respiratory rate in 33 subjects.

### Statistical analysis

Statistical analysis was conducted by SPSS version 21 (SPSS, IBM, Chicago, IL).

Descriptive statistics in terms of mean, standard deviations, median, and percentiles were demonstrated to all parameters in the study. Normal distributions of the quantitative parameters were examined by Kolmogorov‐Smirnov test, and parametric or nonparametric tests were used as appropriate (i.e., nonparametric tests were used for variables with other than normal distribution). Repeated measures analysis with Bonferroni adjustments for multiple comparisons or nonparametric tests for related samples by Friedman tests with controlling for multiple comparisons were used for differences in the measured variables at three repeats for each volunteer. For each volunteer, the average value from the three repeats was calculated.

Differences between normal and obstructed breathing for each volunteer were tested by paired *t*‐test or Wilcoxon paired tests as appropriate. A value of *P* < 0.05 was considered as significant. For measures with significant differences between normal and obstructed breathing, we calculated the receiver operating characteristic curve (ROC curve) using the ROC curve model of SPSS and calculate the best cutoff using Youden index (Ruopp et al. 2008) to evaluate the specificity and sensitivity of this measure to identify obstructed breathing.

## Results

Forty healthy volunteers were recruited, all of which successfully completed all the tasks. Demographic and baseline physiology data are presented in Table 1. ECG was normal in all study subjects; ECG parameters are presented in Table 2. While breathing through a simulated airway obstruction obstructed the respiratory rate dramatically slowed – from 12.9 ± 3.1 breaths min^−1^ during control to 7.5 ± 2.7 breaths min^−1^ during obstructed breathing (*P* < 0.0001, paired *t*‐test). The ratio of respiratory rate of control over obstructed breathing was 1.8 ± 0.45.

**Table 1:** Subjects demographic and baseline physiologic data

Gender (Male/Female)	20/20
Age (year)	20–40
Body mass index (kg m^−2)^	23.0 (3.3)
Systolic blood pressure (mmHg)	122 (13)
Oxygen saturation (%)	99 (1)
Smoking history	12/36^1^
Pack years (of volunteers with smoking history only)	6.4 (6.0)^1^
Routine participation in any physical activity	31/36^1^
Routine participation in strenuous physical activity^2^	23/36^1^
Time spent performing physical activity (hours week^−1^)	3.2 (2.6)^1^

Values are presented as either mean (SD) or portion, aside from age which is presented as range.

1 This data was available for only 36 volunteers.

2 Strenuous physical activity defined as over 6 metabolic equivalents (METs).

**Table 2:** Baseline electrocardiographic data

Heart rate (BPM)	68.6 (10.4)
PR interval (msec)	148 (17)
QT interval (msec)	373 (28)
QTc interval (msec)	395 (19)
*R* waves width (msec)	89 (14)

Values are presented as mean (SD). BPM, beat per minute; PR interval, time between consecutive *P* and *R* waves on the electrocardiogram; QT, interval –time between consecutive *Q* and *T* waves on the electrocardiogram; QTc, corrected QT interval; *R* waves width, time interval between the beginning and end of a *R* waves complex on the electrocardiogram.

A representative example of the R‐R intervals dynamics starting with normal unobstructed breathing and switching to breathing through a simulated airway obstruction is presented in Figure 1B. When comparing each control period (1 min of normal unobstructed breathing) to the following obstructed breathing period (1 min of forced breathing through a 4 mm internal diameter tube), a significant difference can clearly be noticed (Fig. 1B).

In order to ascertain the robustness of the data, every volunteer consecutively performed three such sets (Fig. 1A). For each of the measured variables, we compared the values obtained during the three sets to identify any trend that could bias the repeated task (e.g., learning, adaptation, or not enough time to recover between trials). For most variables, no significant difference could be found between the three sets, suggesting that the recovery interval between trials was adequate, and that no significant fatigue, learning, or adaptation occurred. The average R‐R interval SD of the third set was shorter than that of the second (67.6 ± 37.5 and 67.4 ± 33.1 vs. 59.9 ± 32.6, first, second, and third sets, respectively, *P* = 0.041 for second vs. third, nonsignificant values for first vs. second and first vs. third, Bonferroni adjusted repeated measures analysis of variance). The pNN50 displayed a gradual decrease with repeats from 29.3 ± 22.5 on the first set to 26.8 ± 20.1 on the second and 23.6 ± 19.8 on the third (*P* = 0.026 for first vs. third, nonsignificant values for first vs. second and second vs. third, Bonferroni adjusted repeated measures analysis of variance). Since only two variables showed any significant differences between the three sets and the differences were small and of a limited clinical significance (especially compared to the changes seen during obstructed breathing), we used the subjects’ average values for further analysis.

### Time domain analysis

The mean R‐R interval decreased from 893 ± 115 msec during the control period to 827 ± 100 msec during obstructed breathing (*P* < 0.0001, paired *t*‐test), demonstrating a higher heart rate during obstructed breathing. The average SD of R‐R intervals in a single patient (that should not be confused with the SD of the average R‐R intervals calculated across patients) increased by 66% during obstructed breathing (*P* < 0.0001, paired *t*‐test), and the RMSSD increased by 34% (*P* = 0.001, paired *t*‐test) during that period. NN50 almost doubled during obstructed breathing (25.3 ± 12.3) compared with control (16.5 ± 11.6; *P* < 0.0001, paired *t*‐test) as did the pNN50: 35.1 ± 18.8% during obstructed breathing compared with 26.6 ± 19.9% during control (*P* = 0.001, paired *t*‐test). Time domain values are detailed in Table 3. It can be seen that all time domain variables significantly increased during obstructed breathing, demonstrating the robustness of this finding.

**Table 3:** Time domain values in normal and obstructed breathing

	Normal (*n* = 40)	Obstructed (*n* = 40)	*P* value (paired *t*‐test)	ROC area	Sensitivity (to detect obstruction) (%)	Specificity (for obstruction) (%)
R‐R interval (msec)	893 (115)	827 (100)	<0.0001	0.67^2^	53	80
R‐R Standard Deviation (msec)	65 (32)	108 (41)	<0.0001	0.80^3^	72.5	77.5
RMSSD (msec)	61 (42)	82 (45)	0.001	0.66^1^	50	80
NN50 (events)	16.5 (11.6)	25.3 (12.35)	<0.0001	0.70^2^	85	45
pNN50 (%)	26.6 (19.9)	35.1 (18.8)	0.001	0.63^1^	82.5	43

Values are presented as mean (SD). Sensitivity and specificity were calculated choosing the optimal threshold according to youden model. R‐R interval, time between two consecutive *R* waves on the electrocardiogram; RMSSD, root mean square of successive differences between adjacent R‐R intervals; NN50, number of pairs of successive R‐R intervals that differ by more than 50 msec; pNN50 , proportion of NN50 divided by total number of R‐R intervals.

1 *P* < 0.05 (asymptotic significance compared to *x* = *y*).

2 *P* < 0.01 (asymptotic significance compared to *x* = *y*).

3 *P* < 0.001 (asymptotic significance compared to *x* = *y*).

### Frequency domain analysis

**Figure 2:**
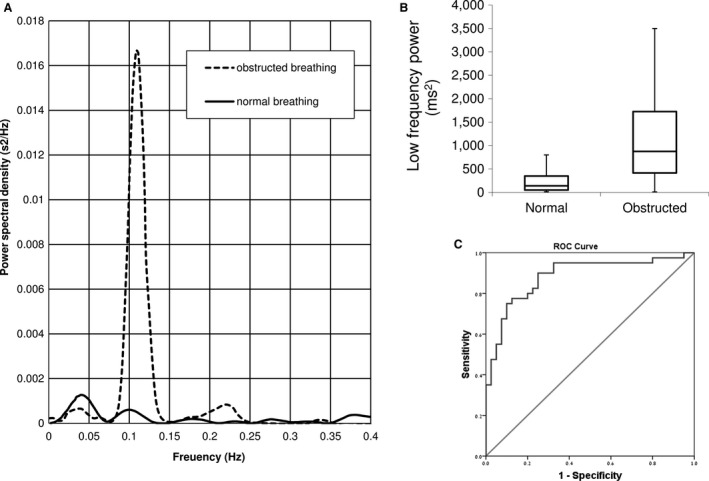
Change in the frequency content of HRV during airway obstruction. A. An example of FFT of the R‐R intervals over one minute of control (solid line), and the immediately following obstructed breathing period (dashed line). The dramatic increase in the LF power can be easily seen. B. The distribution of total LF power during normal breathing (left) and obstructed breathing (right). The horizontal line represents the median; the boxes are the 25th ‐ 75th percentiles. C. Receiver operating characteristic curve calculated for the LF power. Abbreviations: msec‐ millisecond, Hz ‐ Hertz. FFT ‐ Fast Fourier transform, HRV ‐ heart rate variability, LF ‐ low frequency band (0.04‐0.15 Hz)..

Figure 2A shows an example of the FFT of the heart rate during 1 min of control period and a consecutive obstructed breathing period. The increase in the power spectral density of the LF during obstructed breathing can be easily identified. This effect was significant in both the absolute power and the power ratio (the percentage of power spectral density for LF out of the total power spectral density).

### State separation

For all measures with significant differences between normal and obstructed breathing we calculated the ROC curves (see Methods). We evaluated the specificity and sensitivity of each measure to identify obstructed breathing using youden index (Ruopp et al. 2008). The area under the ROC curve was significantly different from the asymptote for most measure (all but HF). The area under the curve, and the calculated sensitivity and specificity to identify airway obstruction are detailed in tables 3 and 4. It is worthwhile to note that the raw LF, LFnorm, and HFnorm all yield sensitivity of 90% or more with a reasonable specificity, suggesting that these measures would be useful monitor to screen for airway obstruction.

**Table 4:** Frequency domain values in normal and obstructed breathing

	Normal (*n* = 40)	Obstructed (*n* = 40)	*P* value (Wilcoxon paired test)	ROC area	Sensitivity (to detect obstruction)	Specificity (for obstruction)
LF power (msec^2^)	164.7 (99.7–314.5)	893.3 (518.3 –1700.0)	<0.0001	0.88^1^	90%	75%
LF norm	56.4 (45.7–74.9)	89.0 (78.0–92.9)	<0.0001	0.85^1^	92.5%	65%
HF power (msec^2^)	105.4 (38.6–251.7)	103.0 (44.8–250.7)	0.51	0.52	–	–
HF norm	38.0 (21.4–48.7)	9.5 (5.1–20.1)	<0.0001	0.86^1^	92.5%	70%

Values are presented as median and 25–75th percentiles. Sensitivity and specificity were calculated choosing the optimal threshold according to youden model. LF, low frequency (0.04‐0.15 Hz); HF, high frequency (0.15–0.4 Hz); LF norm, Normalized LF power; HF norm, Normalized HF power.

1 *P* < 0.001 (asymptotic significance compared to *x* = *y*).

## Discussion

The primary aim of this study was to assess whether HRV can identify airway obstruction accurately and rapidly enough to be useful for monitoring purpose. To do this we used a simulated acute airway obstruction model to show acute changes in HRV in response to obstruction in a controlled, reproducible way. To the best of our knowledge, this has not been previously done. We demonstrated a robust increase in HRV during airway obstruction. The increase was rapid, easy to detect and noteworthy even during a short, 1‐min heart rate sampling period immediately following the onset of the obstruction.

Many factors modulate HRV: psychiatric (Servant et al. 2009), psychologic (Cohen et al. 1998; Vistisen et al. 2014), central nervous system (Kim et al. 2013), anesthesia (Matchett and Wood 2014), body position (Montano et al. 1994; Lowenstein et al. 2014), cardiac preload and afterload, cardiovascular and pulmonary‐respiratory elements, hormones and medications (Weissman et al. 2009; Ulanovsky et al. 2014). HRV analysis was investigated for diagnosis of myocardial ischemia (Goldkorn et al. 2015), and predicting survival in congestive heart failure (Ho et al. 1997) and successful weaning from mechanical ventilation (Huang et al. 2014). Previous studies suggested that LF power of HRV reflects cardiac baroreflex function (Moak et al. 2009; Rahman et al. 2011) and proposed power is modulated by the sympathetic and parasympathetic systems (Reyes del Paso et al. 2013).

The respiratory and cardiac systems are closely related. The cardiorespiratory coupling involves reciprocal interactions mediated partially via the autonomic nervous system (Baselli et al. 1994; Dick et al. 2014; Vidigal et al. 2016). HRV is affected by the respiratory system (Elghozi et al. 1991; Widjaja et al. 2014; Vidigal et al. 2016). Slow and controlled breathing increased the cardiorespiratory coupling as well as LF power and LF/HF ratio (Widjaja et al. 2014; Vidigal et al. 2016). During the period of obstructed breathing, we noticed a slowing of the respiratory rate in our subjects. This slowing of the respiratory rate may modulate HRV. On the other hand, this change is part of the physiologic response to partial airway obstruction, and thus it is part of the change we are trying to identify. In any case, the change we observed in HRV (especially the change in the LF) was dramatically larger than the effect previously observed during slow breath byVidigal et al. (2016), suggesting a separate effect of the obstruction. During obstructed breathing we noticed an increase in some HRV parameters that may be related to autonomic (vagal) activity (e.g., increased pNN50). However, it seems that these changes were mostly related to the obstruction and to the change in intrathoracic pressure, as can be demonstrated by the increase in the heart rate that would not happen has these changes been related to increased vagal activity.

As HRV is influenced via the cardiorespiratory coupling and thus by the respiratory system, it is not surprising that changes in HRV were found to be associated with conditions that impact the airway, especially situations that obstruct the airway. During airway obstruction, intrathoracic pressure rapidly rises and drops as part of the respiratory efforts. This change in the intrathoracic pressure modulates cardiac preload (Konecny et al. 2014). Moreover, such conditions are often accompanied by sympathetic activation which may also impact HRV. Decreased LF and increased HF were reported in asthma patients (Garrard et al. 1992; Gupta et al. 2012) and correlated with the response to methacholine bronchial challenge (Pichon et al. 2005). HRV is modulated by other disorders of dynamic airway obstruction such as OSA (Khoo et al. 1999; Gula et al. 2003; Kufoy et al. 2012; Brzecka et al. 2015), and was even suggested as a diagnostic tool for OSA (Atri and Mohebbi 2015). Recently, Arai et al. (2007) demonstrated that HRV rapidly responds to airway obstruction and to airway opening maneuvers in anesthetized children, demonstrating the potential diagnostic value of HRV for monitoring and rapidly identifying critical airway events under anesthesia. However, they used 5 min segment to calculate HRV, which is too long for monitoring purposes. Our results confirm that the changes in HRV during airway obstruction occurred rapidly and could easily be identified shortly after the airway became obstructed. Moreover, HRV can identify the obstruction with good sensitivity and reasonable specificity Combining the results of Arai et al. (2007) with our study suggest that such a monitoring approach would be highly effective.

To simulate airway obstruction, we used a fixed symmetric obstruction model (equal resistance to inspiration and expiration). This does not necessarily represent all types of airway obstructions, as some causes of obstruction involve a dynamic component, for example, asthma (Lavoie et al. 2012). Our model reflects other common causes encountered in the operating rooms and intensive care units, for example, kinked and small diameter endotracheal tube, or secretions clogging the airway or the tube. Another weakness is that the volunteers were fully awake and without any sedation or anesthesia. It was shown that HRV is sensitive to cortical activity as reflected by psychological conditions (Lane et al. 2009), especially stress (Delaney and Brodie 2000; Hernando et al. 2016; Verkuil et al. 2016). Thus, our airway obstruction simulation could induce stress levels higher than those encountered under deep sedation or anesthesia. The mental effort of maintaining a tight seal of the lips around the breathing tube during obstructed ventilation may also contribute to HRV. However, we do not believe that this is an issue, as none of our patients reported any such stress, they were all aware of the temporary nature of the obstruction and could abort the trail at any time by simply opening their mouth. Moreover, we noticed a fourfold increase in the LF power, which is an order of magnitude larger than the increase reported during stress (Delaney and Brodie 2000; Hernando et al. 2016). Thus, our results cannot be explained simply by the stress response during the obstructed breathing. Further study during anesthesia is required to fully evaluate the contribution of stress response to the increase in LF power. Another limitation is that the study was performed on young healthy volunteers. Extrapolating from this population to older, sicker populations, and especially to patients with cardiac dysrhythmias may be problematic, and will require further experiments.

HRV is usually evaluated over periods much longer than 1 min. The task force of the European Society of Cardiology and the North American Society of Pacing and Electrophysiology recommended 5 min as a minimal ECG recording time for short‐term HRV measurements (Task Force of the European Society of Cardiology and the North American Society of Pacing and Electrophysiology, 1996). However, they did allow the possibility for shorter measurement times in the cases where the nature of the study dictates it. We chose to investigate the effects of HRV during 1 min to test the use of this modality for anesthesia and critical care patients, looking for critical events of airway obstruction. These types of patients and events require a monitor with fast response time (i.e., short data acquisition time) to convey the information before major complications develop. Indeed, most HRV parameters that were calculated using a brief ECG segment of 1 min (including both time and frequency domain parameters) were significantly affected by our acute airway obstruction simulation. Airway obstruction seems to have a dramatic effect on the HRV, enough to allow rapid detection within a shorter than standard sampling time. The brief (1 min) sampling time also allowed us to study awake volunteers, without having to worry about drop out due to the unpleasant nature of breathing through an obstruction for a longer period and raised CO_2_ levels.

We refrained from using VLF frequencies, as VLF includes frequencies below 0.04 Hz, thus a sample of 1 min will include only two periods (or less) of the sampled signal within this range, limiting the accuracy and reliability of the measurements. However, we do believe that the LF results are reliable, and that this frequency range is usable for monitoring even for a short, 1‐min window. Indeed, 1 min includes only two cycles of the lowest frequencies in this range. However, for most of the range there is much more data (up to ten cycles), allowing reliable calculation of the power. Moreover, in our data the differences between the control and obstructed breathing were clear and significant attesting to the usefulness of this measure.

We have shown that a brief period of HRV measurement can be a useful indicator for airway obstruction. This could be a useful addition to our armamentarium of breathing monitors, as this is a cheap, noninvasive modality that can easily be implemented in all current monitors and seems to have a high diagnostic yield. This monitoring technique may be useful during sedation and anesthesia, when spontaneous ventilation is employed. In such cases, the end tidal CO_2_ sampling cannula may dislodge and lead to false alarms. Moreover, this technique may detect instances where air flow persists despite partial obstruction, leading to increased work of breathing and decreased ventilation with nothing to alarm the provider (Ebert et al. 2017).

It has to be noticed however, that this was done in healthy, awake, and spontaneously breathing volunteers. To validate the effectiveness and usefulness of this tool for intraoperative monitoring, further studies are necessary. Such studies include assessment of HRV changes in varying degrees and types of obstruction, correlating the changes to the respiratory effort and performing this under changing levels of anesthesia and sedation.

In conclusion, the use of HRV analysis and especially the LF power calculation from 1‐min samples of raw ECG recording may provide valuable information regarding the respiratory effect on the cardiac cycle, and potentially identify significant airway obstruction. This measure can become a useful adjunct to monitoring end tidal CO_2_.

## Conflict of Interest

A.L., N.G., R.L., and Y.K. declare no competing interests. A.R. Served as a consultant to Medtronic (unrelated to the current project). Presentation: Preliminary results from this work were presented as a poster at the Annual meeting of the Israeli Society of Anesthesiologists in Tel‐Aviv, Israel (15‐16 December 2016).

## Clinical trial number

**Trial number:** NCT03733704.
